# Etiology and severity of various forms of ocular war injuries in patients presenting at an Army Hospital in Pakistan

**DOI:** 10.12669/pjms.326.11158

**Published:** 2016

**Authors:** Syed Abid Hassan Naqvi, Sidra Malik, Syed Zulfiqaruddin, Syeda Birjees Anwar, Shahzad Nayyar

**Affiliations:** 1 Dr. Syed Abid Hassan Naqvi, FCPS, Department of Ophthalmology, Combined Military Hospital, Peshawar, Pakistan; 2 Dr. Sidra Malik, FCPS, Department of Ophthalmology, Combined Military Hospital, Peshawar, Pakistan; 3 Dr. Syed Zulfiqaruddin, FCPS, Department of Ophthalmology, Combined Military Hospital, Peshawar, Pakistan; 4 Dr. Syeda Birjees Anwar, FCPS. Armed Forces Institute of Pathology, Peshawar, Pakistan; 5 Dr. Shahzad Nayyar, Dept. of ENT & Head and Neck Surgery, Combined Military Hospital, Peshawar, Pakistan

**Keywords:** Ocular War Injuries, Open Globe, Closed Globe, Ocular Trauma Score

## Abstract

**Objective:**

To determine the etiology and severity of various forms of ocular war injuries in patients presenting at an Army Hospital in Pakistan.

**Methods:**

This cross sectional study was conducted at the Department of Ophthalmology, Combined Military Hospital, Peshawar over four years period from June 2012 through March 2016, Two hundred ten consecutive soldiers who presented with ocular war injuries were included for analysis after taking written informed consent. A predesigned proforma was used to record patient’s demographic details along with the cause, side, type and severity of injury, ocular trauma score was also recorded at presentation.

**Results:**

The mean age of the patients was 29.34±5.35 years. All of them were males. Left side was more frequently involved (n=126, 60.0%) and the most frequent underlying cause was IED blast injury (n=114, 54.3%). Closed globe injuries were more frequent and were recorded in 120 (57.1%) patients. Upon assigning Ocular Trauma Score, Grade-V (28.6%) injuries were the most frequent followed by Grade-I (25.7%), Grade III (25.7%), Grade II (11.4%) and Grade IV (8.6%). When stratified for the type of injury, OTS Grade I injuries were highest (60.0%) among patients with open globe injuries, hence poorer prognosis, while OTS Grade V injuries were highest (50.0%) among patients with closed globe injuries (p=0.000).

**Conclusion:**

IED blast injuries are most frequently encountered ocular war injuries often involving soldiers in the age group 20-30 years. These open globe injuries had worst clinical presentation to begin with and poorer prognosis than closed globe injuries.

## INTRODUCTION

Ocular trauma is common and causes significant ocular morbidity both in children and adults. Road traffic accident (RTA) is a frequent cause of ocular trauma in general adult population^1^ accounting for one million cases of blindness and 500,000 new cases of unilateral vision loss each year.^2^ However, warfare injuries are becoming a leading cause of vision loss among individuals involved in security forces where the protective clothing does protect the vital organs; chest and abdomen yet leave the face exposed and at risk of injury.^3^ The eyes comprise only 0.27% of the anterior body surface but are frequently affected in blast injuries disproportionate to their size. Previously, before the 20^th^ century, ocular trauma was only four times of its expected percentage of body surface area alone. Due to new explosives with higher fragmentation, this proportion of ocular involvement has increased to over 50 times the expected percentage.^4^-^6^ Thus there has been a gradual increase in the frequency of ocular war injuries from 2% in the 2^nd^ world war to 3% in Korean and 7% in Arab-Israel conflict.^6^-^8^

Pakistan owing to its critical geographical location is involved in a number of security operations including but not limited to those involving American forces at Afghanistan and those with in the country itself against various tribes and extremist movements.^9^ Throughout these operations, Combined Military Hospital Peshawar received a larger proportion of injured army men either directly or after referral from other centers. The present study is an epidemiological analysis of ocular trauma among such patients.

## METHODS

This was a cross sectional study conducted at the Department of Ophthalmology, Combined Military Hospital, Peshawar over four years period from June 2012 through March 2016. During, this period, 210 consecutive soldiers who presented with ocular war injuries were included for analysis after taking written informed consent. Patients who were dead on arrival or those who expired within 24 hours of presentation where examination could not be completed were excluded from this study. A predesigned proforma was used to record patient’s demographic details along with the cause, side, type and severity of injury. Injuries were classified as open globe or closed globe injuries. Ocular Trauma Score Grade was calculated and used to describe the severity of injury. All the patients were assessed by a single consultant ophthalmologist to eliminate bias.

## RESULTS

The age of the patients ranged from 20 years to 43 years with a mean of 29.34±5.35 years. Majority (n=156, 74.3%) of the patients were aged between 20-31 years. All of them were male. Left side was more frequently involved (n=126, 60.0%). The most frequent underlying cause was IED blast injury (n=114, 54.3%) followed by blunt trauma (n=42, 20.0%) and RTA (n=24, 11.4%). Closed globe injuries were more frequent and were recorded in 120 (57.1%) patients.

When stratified the nature of injury, there was no significant difference with the patient’s age (p=0.784) and side involved (p=1.000). However, IED blast (73.3%), splinter (13.3%) and GSW (13.3%) were the frequent causes observed in patients with open globe injuries while IED blast (40.0%), blunt trauma (35.0%) and RTA (20.0%) were the frequent cause observed in patients with closed globe injuries. The observed difference was statistically significant (p=0.000) as shown in Table-I.

**Table-I:** Stratification of Nature of injury across patient’s age, side involved and underlying cause.

Characteristic	Open Globe Injuries n=90	Closed Globe Injuries n=120	P value
*Age Groups*			
• 20-31 years	66 (73.3%)	90 (75.0%)	0.784
• 32-43 years	24 (26.7%)	30 (25.0%)	
*Side*			
• Right	36 (40.0%)	48 (40.0%)	1.000
• Left	54 (60.0%)	72 (60.0%)	
*Cause of Injury*			
• IED Blast	66 (73.3%)	48 (40.0%)	0.000*
• RTA	0 (.0%)	24 (20.0%)	
• Blunt Trauma	0 (.0%)	42 (35.0%)	
• Splinter	12 (13.3%)	6 (5.0%)	
• GSW	12 (13.3%)	0 (.0%)	

Chi-square tests,

* observed difference was statistically significant.

On the Closed Globe Injury Classification, majority of the patients had contusion (n=20, 35.0%) followed by mixed type seen in 36 (30.0%) patients. Majority (n=66, 55.0%) of the patients had Grade-I injury followed by Grade-IV injury in 30 (25.0%) patients. Pupil was negative in 108 (90.0%) patients while in majority of the patients injury involved Zone-I (n=84, 70.0%). All these findings have been summarized in Table-II.

**Table-II:** Closed Globe Injury Classification.

Closed Globe Injury Classification	Study Participant n=120
*Type*	
• Contusion	42 (35.0%)
• Lamellar Laceration	18 (15.0%)
• Superficial Foreign Body	24 (20.0%)
• Mixed	36 (30.0%)
*Grade*	
• ≥20/40	66 (55.0%)
• 20/50 – 20/100	12 (10.0%)
• 19/100 – 5/200	12 (10.0%)
• 4/200 – light perception	30 (25.0%)
• No perception of light	-
*Pupil (RAPD)*	
• Positive	12 (10.0%)
• Negative	108 (90.0%)
*Zone*	
• I	84 (70.0%)
• II	24 (20.0%)
• III	12 (10.0%)

On the Open Globe Injury Classification, penetrating injury (n=48, 53.3%) was the most common followed by intraocular foreign body (n=24, 26.7%). It was Grade-IV injury in majority of cases (46.7%) followed by Grade-V injury (33.3%). Pupil (relative afferent pupillary defect) was negative in all the patients (100%) with open globe injury. Zone II and Zone III were equally involved and were seen in 40% of patients with open globe injuries (Table-III).

**Table-III:** Open Globe Injury Classification.

Open Globe Injury Classification	Study Participant n=90
*Type*	
• Rupture	6 (6.7%)
• Penetrating	48 (53.3%)
• Intraocular Foreign Body	24 (26.7%)
• Perforating	6 (6.7)
• Mixed	6 (6.7%)
*Grade*	
• ≥20/40	-
• 20/50 – 20/100	-
• 19/100 – 5/200	18 (20.0%)
• 4/200 – light perception	42 (46.7%)
• No perception of light	30 (33.3%)
*Pupil (RAPD)*	
• Positive	-
• Negative	90 (100%)
*Zone*	
• I	18 (20.0%)
• II	36 (40.0%)
• III	36 (40.0%)

Upon Ocular Trauma Score, Grade-V (28.6%) injuries were the most frequent followed by Grade-I (25.7%), Grade III (25.7%), Grade II (11.4%) and Grade IV (8.6%). When stratified for the type of injury, OTS Grade I injuries were highest (60.0%) among patients with open glob injuries while OTS Grade V injuries were highest (50.0%) among patients with closed globe injuries. The observed difference was statistically significant (p=0.000) as shown in Table-IV.

**Table-IV:** Ocular Trauma Scale Grades.

Ocular Trauma Scale Grades	Overall	Open Globe Injuries n=90	Closed Globe Injuries n=120	P value
Grade-I	54 (25.7%)	54 (60.0%)	0 (.0%)	
Grade-II	24 (11.4%)	12 (13.3%)	12 (10.0%)	0.000
Grade-III	54 (25.7%)	24 (26.7%)	30 (25.0%)	
Grade-IV	18 (8.6%)	0 (.0%)	18 (15.0%)	
Grade-V	60 (28.6%)	0 (.0%)	60 (50.0%)	

Chi-square tests, *observed difference was statistically significant.

## DISCUSSION

Ocular injuries put socio-economic burden over the society in both short and long term consequences. These can be primary when they result from blast wave itself, secondary caused by blast fragments, tertiary due to structural collapse or by push against an object, or quaternary due to burns or other indirect injuries.^10^

In the present study, the most frequent underlying cause of war related ocular injuries was IED blast followed by blunt trauma and RTA. These injuries were frequently closed globe and fell under Ocular Trauma Score Grade-V. OTS Grade I injuries, with a marked poorer prognosis, were highest among patients with open globe injuries while OTS Grade V injuries, with relatively better prognosis, were highest among patients with closed globe injuries. The observed difference was statistically significant (p=0.000). Nadeem et al. in another local study reported similar mean age of 23.3±17.3 years with male predominance (m:f 4.92:1) among patients presenting with ocular injuries at Ophthalmology Department Fauji Foundation Hospital, Rawalpindi. They reported relatively higher frequency of right eye involvement (63.9%).^11^ They however included all kind of ocular injuries not limited to warfare injuries as compared to the present study.

Among Indian population, Shashikala et al. observed 20-30 years being the most frequently involved age group and closed globe injuries being most frequent (94.4%) at an industrial hospital.^12^ Boparai et al. also observed IED blast injury to be the most frequent cause with gradual increase over time; 71% in 1965 to 76% in 1971.^4^ Mader et al. reported open globe injuries to be more frequent (63.77%) and IED blast to be the most frequent underlying cause observed in 51% of all ocular war injuries.^13^

Weichel et al. observed a similar mean age of 25±7 years among soldiers involved in Iraq war presenting with ocular war injuries with male predominance (96.0%). In line with the present study, they too reported IED being the most frequent underlying cause and observed it in 79% cases.^3^

The observations made in the present study are similar to the observations made by other authors previously suggesting common mechanism, pattern and severity of ocular war injuries. Based on these observations, soldiers presenting with warfare injuries can be suspected of common pattern of ocular injuries and anticipated management in such cases can lead to decrease in early and long term morbidity. Moreover emphasis should be placed on prevention of ocular injures through protective eyewear, not only in war but routine circumstances as well.

## CONCLUSION

IED blast injuries are the most frequently encountered ocular war injuries. Open globe injuries had worst clinical presentation to begin with and poorer prognosis than closed globe injuries. The importance of prevention with protective eyewear cannot be undermined.
